# Mechanochemistry of supramolecules

**DOI:** 10.3762/bjoc.15.86

**Published:** 2019-04-12

**Authors:** Anima Bose, Prasenjit Mal

**Affiliations:** 1 School of Chemical Sciences, National Institute of Science Education and Research (NISER), HBNI, Bhubaneswar, PO Bhimpur-Padanpur, Via Jatni, District Khurda, Odisha 752050, India

**Keywords:** ball milling, mechanochemistry, self-assembly, solvent-free, supramolecular

## Abstract

The urge to use alternative energy sources has gained significant attention in the eye of chemists in recent years. Solution-based traditional syntheses are extremely useful, although they are often associated with certain disadvantages like generation of waste as by-products, use of large quantities of solvents which causes environmental hazard, etc. Contrastingly, achieving syntheses through mechanochemical methods are generally time-saving, environmentally friendly and more economical. This review is written to shed some light on supramolecular chemistry and the synthesis of various supramolecules through mechanochemistry.

## Introduction

In living systems an important aspect is to create complex functional molecules from simpler units by following biomolecular mechanisms [1]. The biological assemblies for living beings are developed from processes of spontaneous self-assembly with a high degree of compartmentalization [2]. In addition, the same building units are often used across an enormous number of structures in a reversible fashion through thermodynamic control [3]. Conversely, small-molecule synthesis is generally performed under kinetically controlled reaction conditions through covalent approaches. By using common synthetic methodologies chemists are able to proficiently synthesize a variety of both natural and unnatural molecular scaffolds [4–6].

The era of supramolecular chemistry began with the introduction of coordination theory by Alfred Werner in 1893 [7] followed by the lock-and-key concept of Emil Fischer in 1894 [8]. Weak or non-covalent interactions had been used systematically in the early 1960s by Lehn, Cram and Pederson to create targeted supramolecular architectures [9]. Small molecules, anions or cations could be assembled spontaneously to form supramolecular structures through self-assembly processes by exploiting the weak or non-covalent interactions [10]. Self-assembly is a kinetically reversible process which is more efficient than traditional stepwise synthesis concerning large molecules. Some recent developments in supramolecular chemistry are dynamic combinatorial chemistry [11], subcomponent self-assembly approach [12–14], and systems chemistry [15–18], etc.

There also has been growing interest towards exploration of nontraditional energy sources like visible light [19–20], microwave [21], mechanochemical mixing [22–23], ultrasound [24], etc. as alternative energy sources to replace common laboratory techniques [25]. Among them, especially mechanochemical synthesis [26–29] has gained popularity due to its advantage over conventional solution-based methods [30]. The process is highly beneficial as the solvent-free condition may make traditional workup superfluous [31–32]. Also, mechanochemical methods have high impact in ecology and economy as they save time [33]. Mechanochemical syntheses benefit from high to quantitative conversions, minimized steps for purification and diminished liberation of undesired byproducts [34–35]. In literature, classical small organic molecules’ synthesis has been well explored which includes multistep synthesis [36–39]. However, the concept of supramolecular chemistry under mechano-milling conditions only has limited number of examples [40–42].

## Review

### Self-assembly

**Figure 1:**
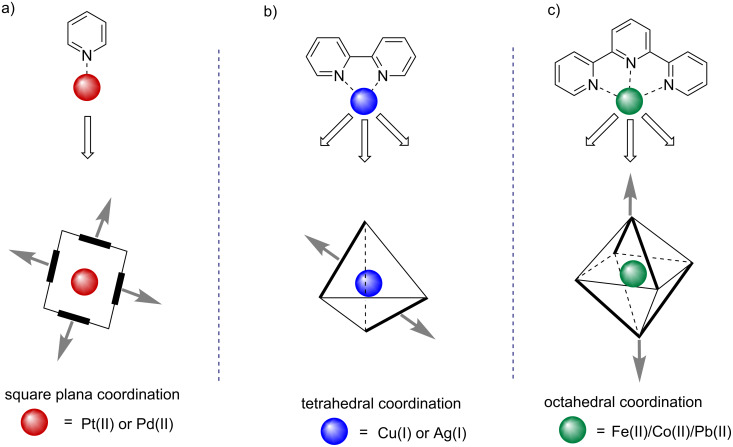
A generalized overview of coordination-driven self-assembly.

During a metal-directed self-assembly process, the coordination geometry and coordination number at the metal center plays a central role in creating a self-assembled system. In 1962, Busch and coworker first demonstrated the concept of the template effect by choosing a suitable metal ion to control a self-assembly process [43–44]. The template enforces the assembly of the smaller units around it in a distinct and organized way favoring the formation of a particular product from a mixture with multiple possibilities [45]. Therefore, the concept of selection of appropriate metal ion(s) and ligand(s) has been demonstrated in various reports [46–49]. In Figure 1, a comprehensive framework is shown in which nanoscale architectures are built from various monodentate (pyridine; Figure 1a), bidentate (bipyridine, phenanthroline; Figure 1b) and tridentate (terpyridines; Figure 1c) ligands [49]. The model systems depicted in Figure 1 are constructed using metals like Pd(II) or Pt(II) ions for square planar geometry, Cu(I) or Ag(I) ions for tetrahedral geometry and Co(II)/Cu(II)/Fe(II)/Zn(II)/Hg(II) for octahedral organization [50].

**Figure 2:**
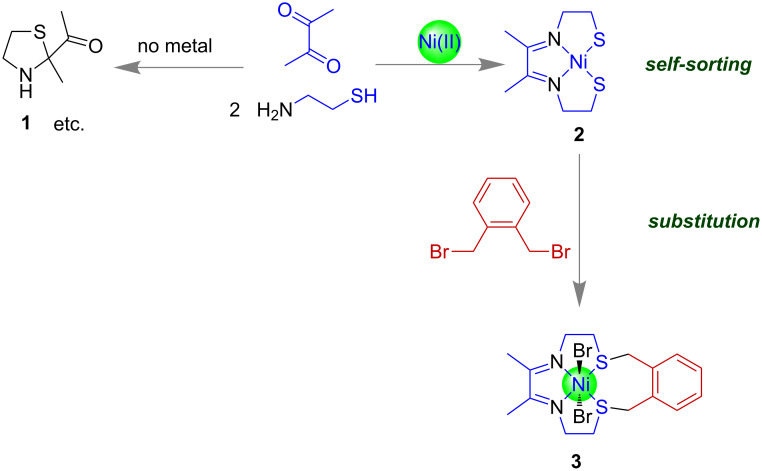
Examples of self-assembly or self-sorting and subsequent substitution.

In Figure 2, Busch’s first example of a metal-directed self-assembly is shown [44]. The mixing of diacetyl and 2-aminoethanethiol led to a dynamic mixture of products including **1**. The square-planar directing metal ion nickel(II) induces the formation of cyclic product **2** through a process called self-sorting [51–53]. Subsequently, compound **2** underwent substitution with α,α’-dibromo-*o*-xylene to create the nickel(II) complex **3** [54].

**Figure 3:**
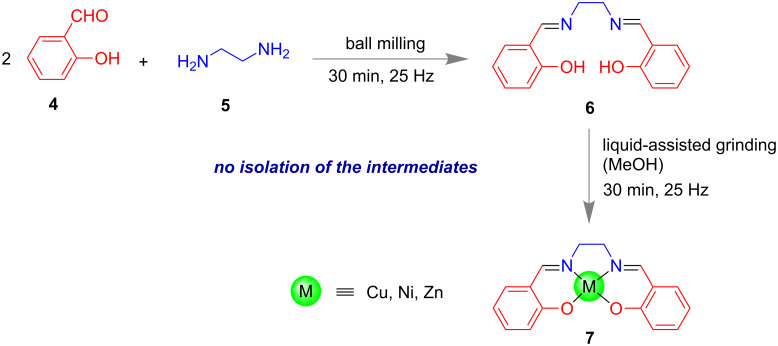
Synthesis of salen-type ligand followed by metal-complex formation in the same pot [55].

In 2014, James and co-workers reported a one-pot two-step mechanochemical synthesis of metal complexes **7** (Figure 3). First, the salen-type ligand **6** was synthesized from *o*-hydroxybenzaldehyde (**4**) and ethylenediamine (**5**). Subsequently, to the same pot appropriate metals were added to obtain the respective complexes **7** [55].

**Figure 4:**
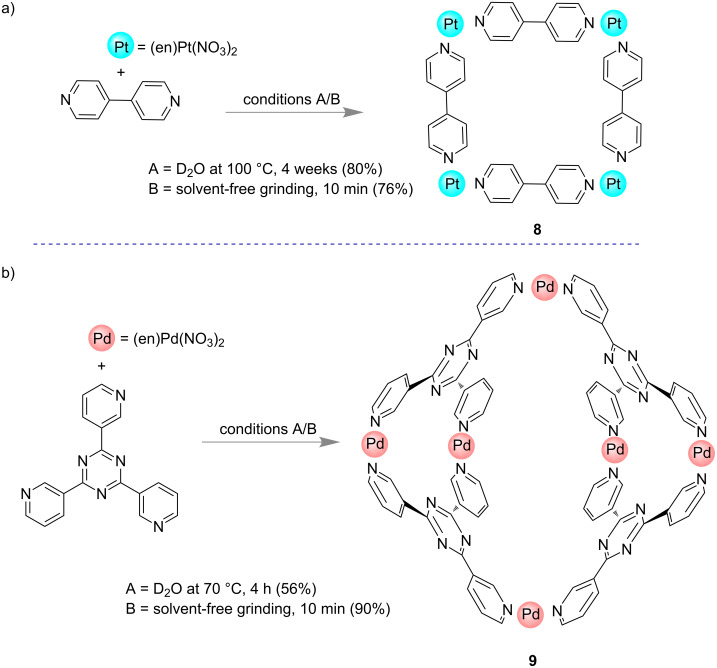
Otera’s solvent-free approach by which the formation of self-assembled supramolecules could be accelerated [56].

In 2002, Otera and co-workers reported the formation of some supramolecular self-assembled structures which was found to be faster under solvent-free mechanochemical condition than in aqueous media [56]. When a 1:1 mixture of the platinum salt [(en)PtNO_3_)_2_] and 4,4'-bipyridine were grinded in a mortar and pestle for 10 min, an NMR yield of 76% was found for the formation of molecular square **8** (Figure 4a). Similar structures were reported by Fujita’s group [57] in which the formation of a Pt-based supramolecular square took more than four weeks at 100 °C. Using a similar approach, Otera’s group also demonstrated for the formation of a bowl-shaped assembly **9** in 90% yield upon grinding for 10 min 2,4,6-tri(pyridin-3-yl)-1,3,5-triazine and palladium ((en)Pd(NO_3_)_2_, Figure 4b). Contrastingly, in solution the same synthesis took 4 h at 70 °C to isolate complex **9** in 56% yield.

**Figure 5:**
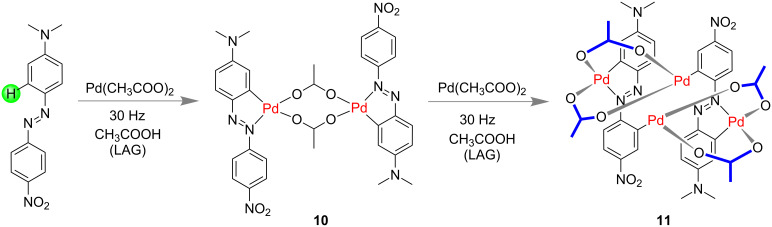
Synthesis of a Pd-based metalla-supramolecular assembly through mechanochemical activation for C–H-bond activation of unsymmetrically substituted azobenzene [58].

Ćurić and co-workers prepared cyclopalladated complexes such as **10** by a grinding method and were the first to confirm a mechanochemical C–H bond activation of an unsymmetrically substituted azobenzene [58]. The cyclopalladation process was proved to be a highly regioselective process and the observed palladation rate was faster compared to the conventional solution-phase method. An equimolar amount of 4'-(*N,N*-dimethylamino)-4-nitroazobenzene and Pd(OAc)_2_ in the presence of 25 μL of glacial acetic acid (for liquid-assisted grinding, LAG) led to the regioselective C–H activation (Figure 5) [58].

**Figure 6:**
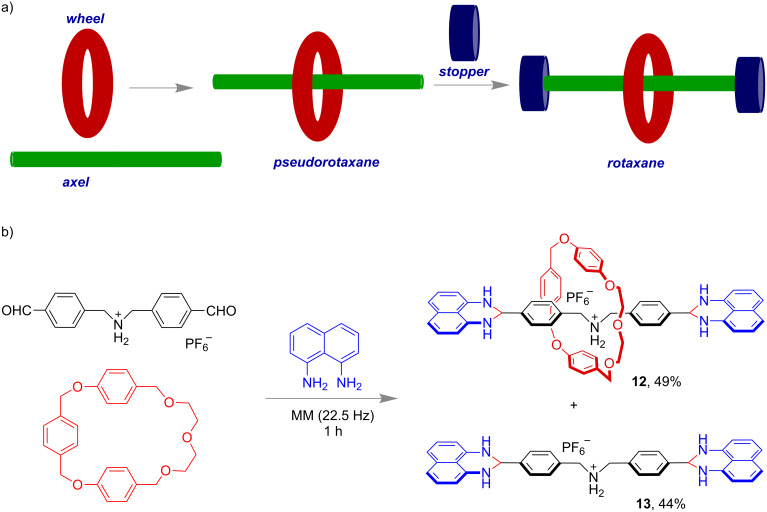
a) Schematic representation for the construction of a [2]rotaxane. b) Chiu’s ball-milling approach for the synthesis of [2]rotaxanes.

In 2008, the mechanochemical synthesis of both [2]- and [4]rotaxanes was reported by Chiu and co-workers. The reactions led to high yields of the products **12** and **13** under solvent-free conditions at a milling frequency of 22.5 Hz (Figure 6b) [59]. The stoppers were constructed in situ with 1,8-diaminonaphthalene through the formation of an imine via dehydration of the amine and aldehyde.

**Figure 7:**
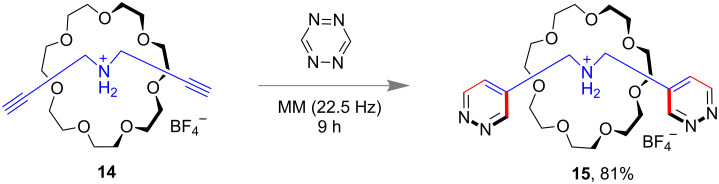
Mechanochemical synthesis of the smallest [2]rotaxane.

Interestingly, a synthesis of the smallest [2]rotaxane also has been demonstrated by the same group [60]. They applied a Diels–Alder reaction of 1,2,4,5-tetrazine with a terminal alkyne unit in a 21-crown-7-based [2]pseudorotaxane **14**. The [2]rotaxane **15** was produced in 81% yield having pyridazine groups as stoppers (Figure 7).

**Figure 8:**
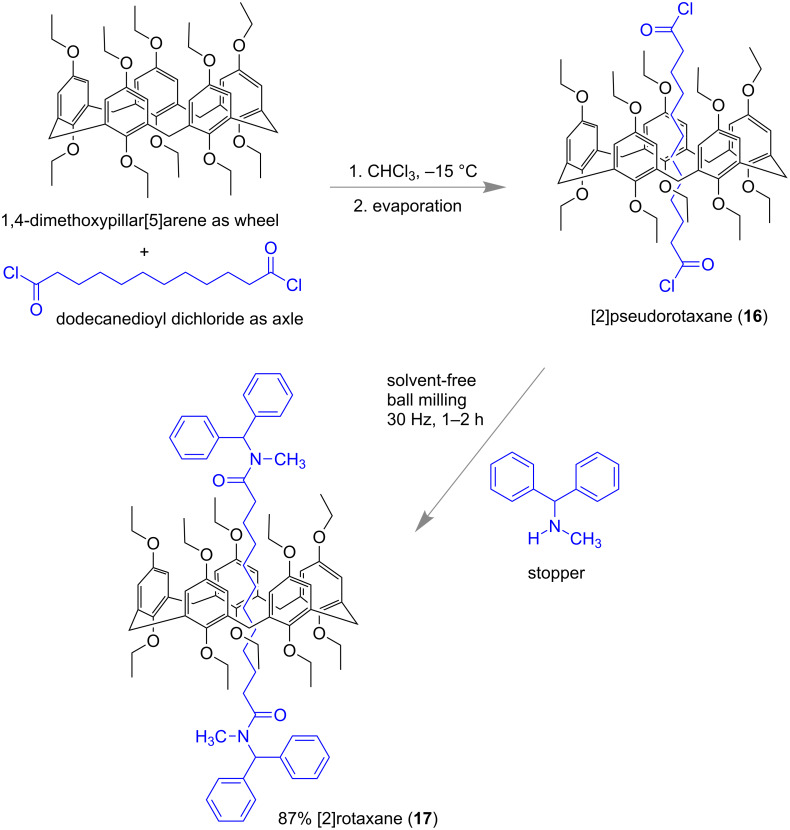
Solvent-free mechanochemical synthesis of pillar[5]arene-containing [2]rotaxanes [61].

Very recently, Nierengarten and co-workers reported a solvent-free mechanochemical synthesis of pillar[5]arene-containing [2]rotaxanes (Figure 8). Mixing a 2:1 ratio of pillar[5]arene (wheel) with dodecanedioyl dichloride (axle) in CHCl_3_ resulted in the formation of pseudorotaxane **16** which was further treated with different amines (stopper) in a stainless-steel jar with 4 steel balls under milling conditions (30 Hz for 1–2 h). When for example, *N*-methyl-1,1,-diphenylmethanamine was used as one of the stoppers, diamido [2]rotaxane **17** was obtained with high yield (ca. 87%) [61] (Figure 8).

**Figure 9:**
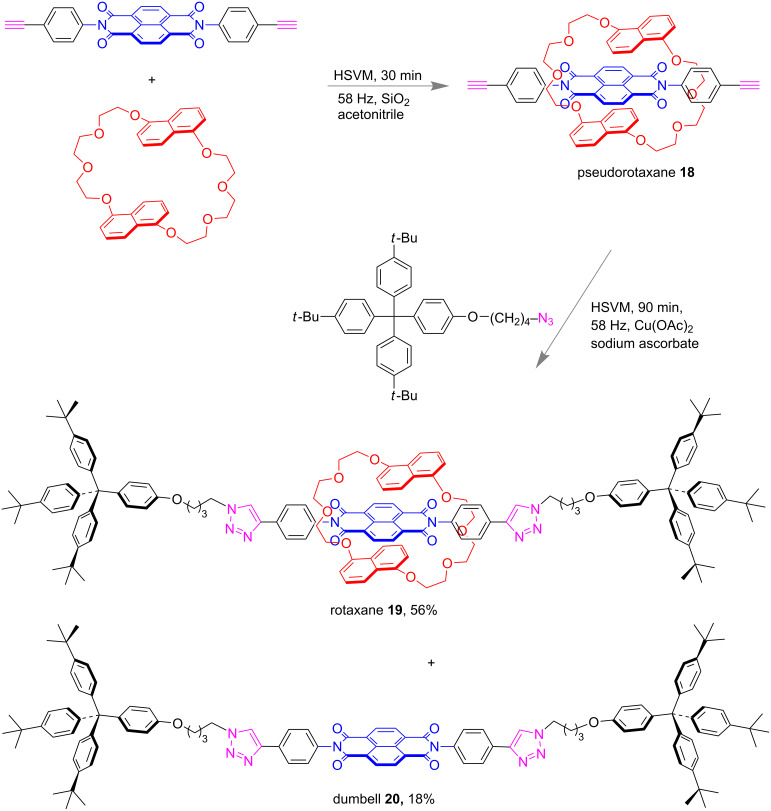
Mechanochemical liquid-assisted one-pot two-step synthesis of [2]rotaxanes under high-speed vibration milling (HSVM) conditions [62].

In 2017, Wang and co-workers reported an efficient method for the synthesis of neutral donor–acceptor [2]rotaxanes such as **19** through liquid-assisted mechanochemical milling (Figure 9). The donor–acceptor interaction between the electron-deficient naphthalene diimide moiety and the electron-rich naphthalene moieties embedded in the macrocyclic polyethers played the vital role for the construction of the rotaxane system via pseudorotaxane **18**. A shorter reaction time, use of small amounts of solvent and the high yield were the advantages over the solvent-mediated synthesis [62].

### Macrocycle synthesis

**Figure 10:**
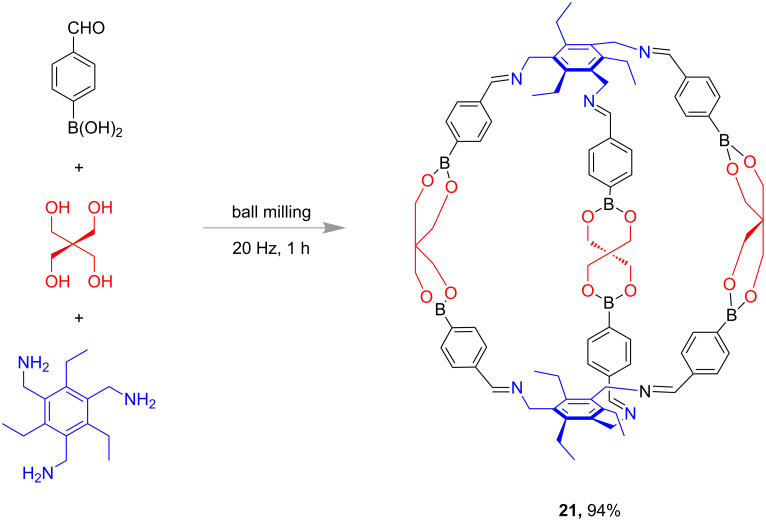
Mechanochemical (ball-milling) synthesis of molecular sphere-like nanostructures [63].

The mechanochemical synthesis of sphere-like nanostructures was reported by Severin and co-workers (Figure 10). Under ball-milling conditions (20 Hz), the condensation of 4-formylphenylboronic acid, pentaerythritol and 1,3,5-tri(aminomethyl)-2,4,6-triethylbenzene afforded 94% of sphere-like compound **21** in 1 h [63].

**Figure 11:**
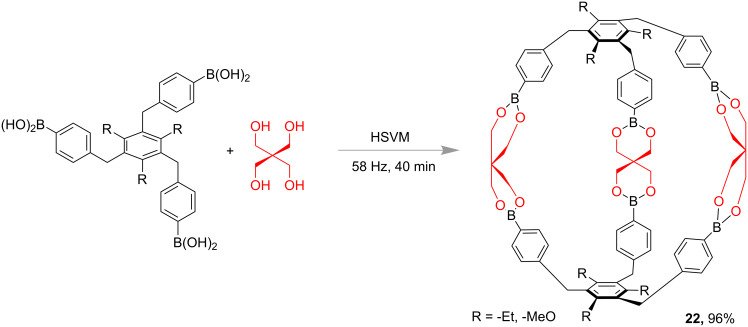
High-speed vibration milling (HSVM) synthesis of boronic ester cages of type **22** [64].

In 2018, Wang and co-workers also demonstrated the synthesis of boronic ester cages under high-speed vibration milling (HSVM) conditions. The condensation of pentaerythritol and triboronic acid at 58 Hz for 40 min led to the formation of cage structure **22** (Figure 11) with nearly 96% yield [64]. The authors also reported that the cage compounds such as **22** had high thermal stabilities by exhibiting a decomposition temperature up to 320 °C.

**Figure 12:**
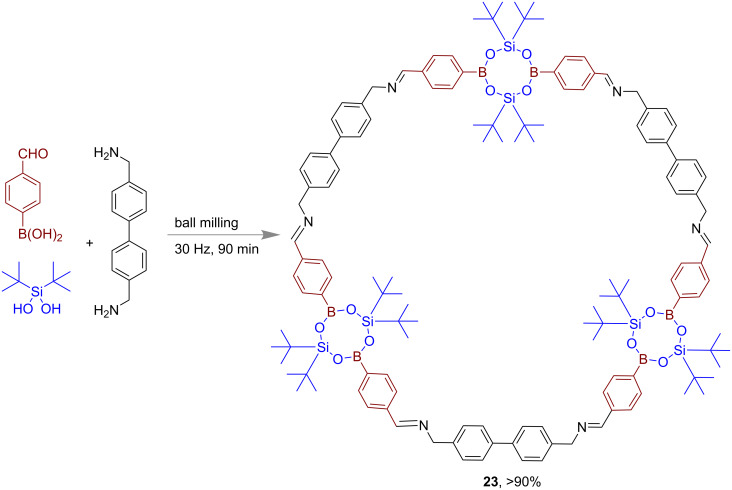
Mechanochemical synthesis of borasiloxane-based macrocycles.

In 2013, Severin and co-workers reported the mechanochemical synthesis of large macrocycles with borasiloxane and imine links using a ball mill (Figure 12). In a polycondensation reaction using diamines, 4-formylbenzeneboronic acid and *t*-Bu_2_Si(OH)_2_, borasiloxane-based macrocycle **23** was obtained in >90% yield after 2 × 45 min of grinding [65].

**Figure 13:**
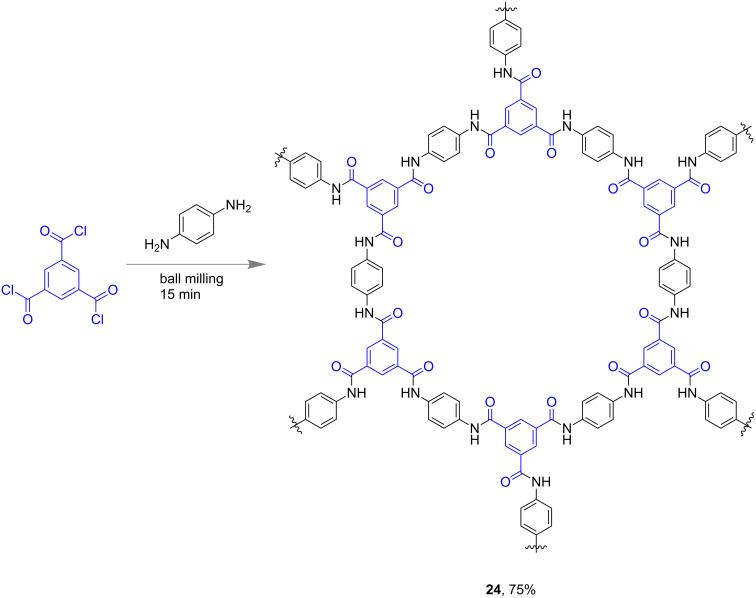
Mechanochemical synthesis of 2-dimensional aromatic polyamides.

In 2017, Xu and his group developed the first method towards the synthesis of 2-dimensional aromatic polyamides (2DAPAs) under solvent-free ball-milling conditions [66]. Reacting 1,3,5-benzenetricarbonyl chloride and 1,4-phenylenediamine in a ball mill afforded 75% of the desired 2D polymer **24** within 15 min (Figure 13). When using 4,4'-diaminobiphenyl in place of 1,4-phenylenediamine the analogous 2D structure comprising biphenylene units was obtained within the same time albeit with a lower yield (≈65%).

### Subcomponent self-assembly and mechano-milling

Nature displays innumerable and beautiful creations [67–68] which include highly complex self-assembled structures made from smaller building blocks by using weak or non-covalent interactions [69–70]. Therefore, the supramolecular approach [71] and systems chemistry [1,72] are considered as the fastest growing areas of chemical research during the last couple of decades [73–74]. The concept of systems chemistry offers a thorough understanding of the building-up principles for creation of complex functional molecular systems from conventional materials [75–76]. The systems chemistry approach may give easy access to new structures or functional materials simply by controlling the inputs of a multicomponent system. The concept of self-sorting [77–79] and subcomponent self-assembly approach [80] are well-developed methods being practiced in supramolecular chemistry to produce complex molecules with topological diversity [81]. Therefore, organic transformations through subcomponent synthesis under mechano-milling conditions might be considered as a useful tool for performing a chemical reaction in a greener fashion.

**Figure 14:**
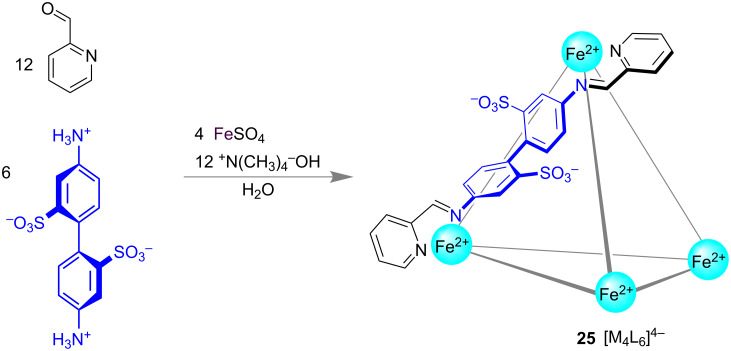
Nitschke’s tetrahedral Fe(II) cage **25**.

The subcomponent self-assembly of a rigid aromatic linear bisamine, pyridine-2-carboxaldehyde and Fe(II) resulting in the tetrahedral [M_4_L_6_]^4−^ cage **25** in water reported by Nitschke [82] was a milestone of supramolecular tetrahedral complex chemistry (Figure 14). The authors have thoroughly explored the host–guest chemistry of that self-assembled Fe(II) cage [4,12,83].

**Figure 15:**
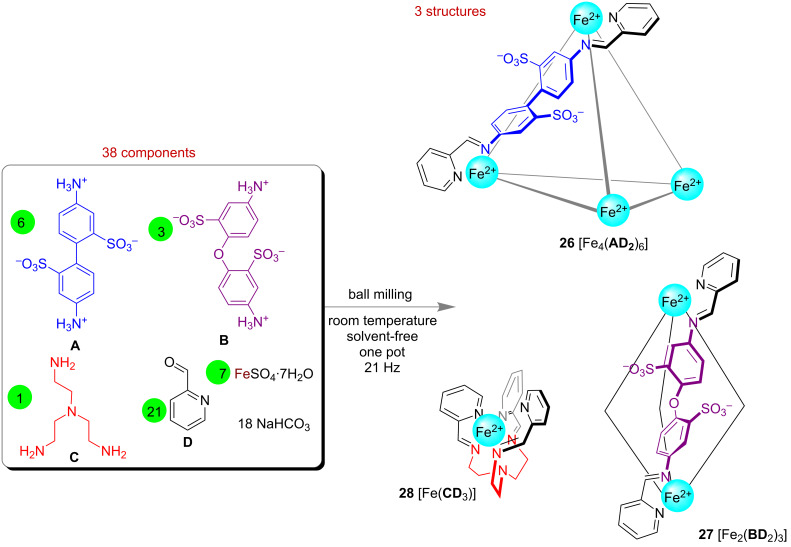
Mechanochemical one-pot synthesis of the 22-component [Fe_4_(**AD**_2_)_6_]^4−^ **26**, 11-component [Fe_2_(**BD**_2_)_3_]^2−^ **27** and 5-component [Fe(**CD**_3_)]^2+^** 28**.

In 2015, Mal’s group successfully reproduced the synthesis of Nitschke’s tetrahedral iron-cage molecule under solvent-free mechano-milling conditions [84]. Subcomponent self-assembly from components **A**, **B**, **C**, **D** and Fe(II) in a solvent-free environment through self-sorting led to three structurally different metallosupramolecular Fe(II) complexes. Under mechano-milling conditions the tetranuclear [Fe_4_(**AD**_2_)_6_]^4−^ 22-component cage **26**, dinuclear [Fe_2_(**BD**_2_)_3_]^2−^ 11-component self-assembled helicate **27** and 5-component mononuclear [Fe(**CD**_3_)]^2+^ complex **28** could be prepared simultaneously in a one-pot reaction starting from 38 components (Figure 15).

**Figure 16:**
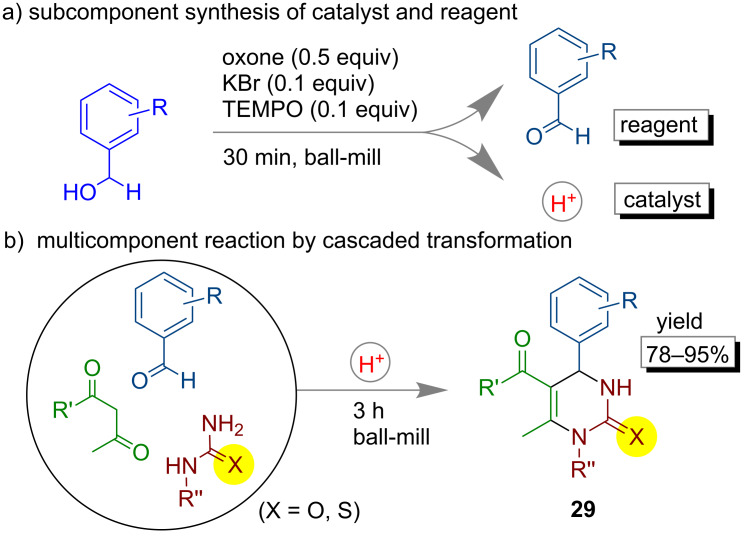
a) Subcomponent synthesis of catalyst and reagent and b) followed by multicomponent reaction for synthesis of dihydropyrimidones.

In 2015, Mal and co-workers described a multicomponent Biginelli [85] reaction following a subcomponent synthesis under mechanochemical conditions. They have developed a method in which dihydropyrimidone synthesis was achieved from benzyl alcohol using a Br^+^ source as the catalyst (Figure 16). In the reaction pot subcomponents such as benzaldehydes and H^+^ were formed which further participated in a cascade transformation to give dihydropyrimidones **29**.

### Dynamic combinatorial chemistry and mechano-milling

**Figure 17:**
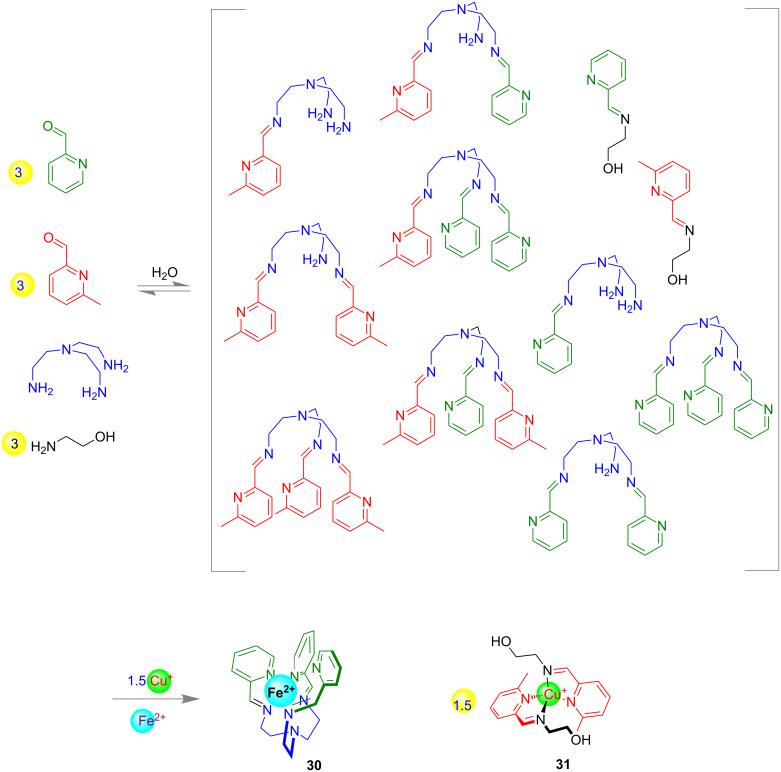
A dynamic combinatorial library (DCL) could be self-sorted to two distinct products.

Dynamic combinatorial chemistry (DCC) is one of the most important topics which make us understand the relationship between complex molecules and living systems. With this approach, a library of chemical species called dynamic combinatorial library (DCL) can be designed which are in thermodynamic equilibrium with each other. Nitschke and co-workers reported, that mixing of 2-formylpyridine (3.0 equiv), 6-methyl-2-formylpyridine (3.0 equiv), tris(2-aminoethyl)amine (1.0 equiv) and ethanolamine (3.0 equiv) in aqueous solution afforded a dynamic library of imines which subsequently could be self-sorted into two distinct complexes **30** and **31** upon the addition of Cu(I) tetrafluoroborate (1.5 equiv) and Fe(II) sulfate (1.0 equiv) as shown in Figure 17 [86].

**Figure 18:**
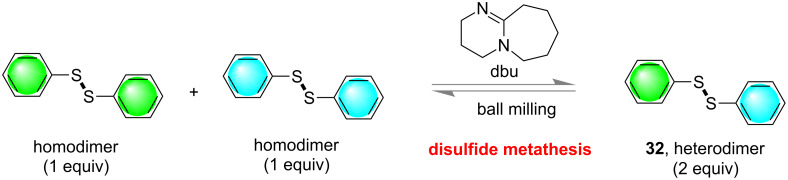
Mechanochemical synthesis of dynamic covalent systems via thermodynamic control.

DCL formation was also shown to be possible in the solid-state by grinding or mechanochemical methods by Sanders and co-workers in 2011. They have demonstrated the reversibility and thermodynamic control in mechanochemical covalent synthesis, towards base-catalyzed metathesis of aromatic disulfides as a model reaction [87]. The outcome of solution-phase chemistry and mechanochemical synthesis were well distinguished and they have described the phenomenon based on differences in the crystal packing in the solid state. The products **32** were obtained via thermodynamic control (Figure 18) from a dynamic combinatorial library [53,88].

**Figure 19:**
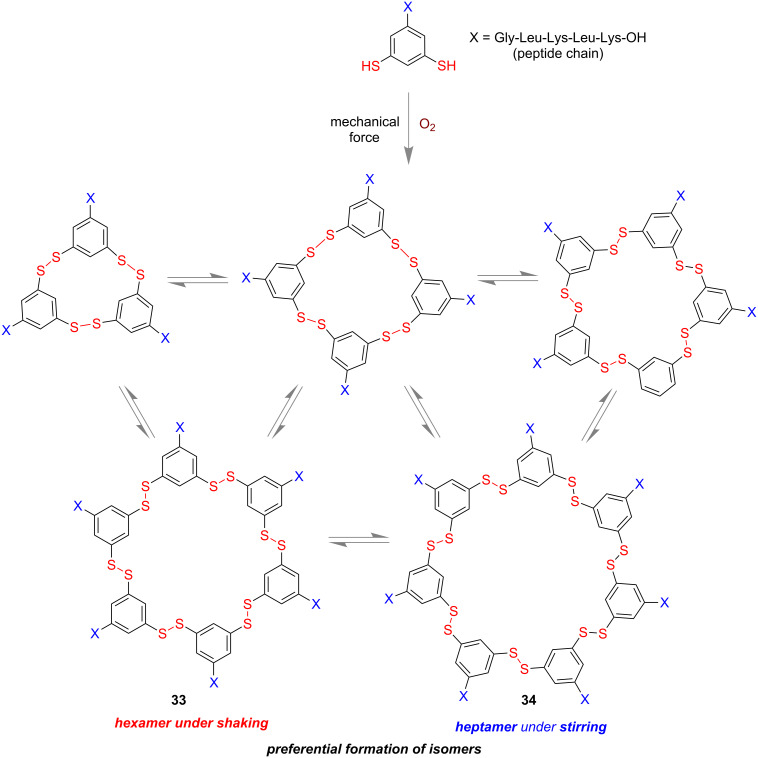
Preferential formation of hexamer **33** under mechanochemical shaking via non-covalent interactions of peptide chains.

In 2010, Otto and co-workers observed unprecedented product selectivity for the formation of disulfide macromolecules based on mechanical shaking and stirring [89]. Peptide-chain containing distal thiol groups underwent an aerial oxidation process to give different disulfide-containing macromolecules. They observed that under mechanical shaking conditions preferentially the cyclic hexamer **33** is formed, whereas stirring resulted in formation of heptamer **34** as the major isomer (Figure 19). From this observation the authors concluded that not only the thermodynamically controlled products but also the kinetically controlled products could be obtained in DCL depending on the non-covalent interactions present in the molecule. Non-covalent interactions of alternating hydrophilic and hydrophobic units in the peptide chains played the vital role in the system.

**Figure 20:**
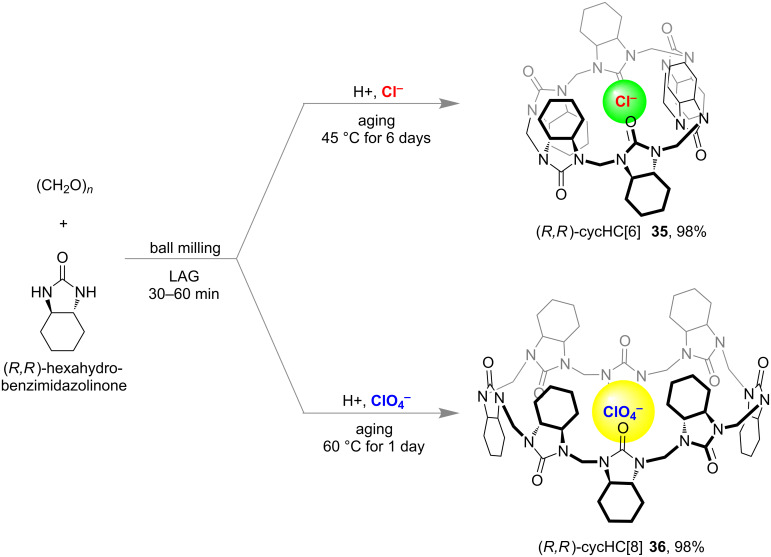
Anion templated mechanochemical synthesis of macrocycles cycHC[*n*] by validating the concept of dynamic covalent chemistry.

Friščić and Aav with co-workers reported the first solvent-free mechanochemical synthesis of hemicucurbiturils [90] through the anion template effect of dynamic covalent chemistry [47,91–92]. The mechanochemical milling of a 1:1 mixture of paraformaldehyde and (*R,R*)-hexahydro-2-benzimidazolinone along with a small amount of concentrated aqueous HCl for 30–60 min followed by aging at 45 °C for 6 days, resulted in the formation of six-membered macrocycle cycHC[6] **35** with 98% conversion by NMR (Figure 20). When ClO_4_^−^ has been used as the anion template, the formation of the eight-membered macrocycle cycHC[8] **36** was observed in 98% conversion by NMR after 30 min of LAG, followed by aging for one day at 60 °C [90].

### Template-assisted mechanochemistry

**Figure 21:**
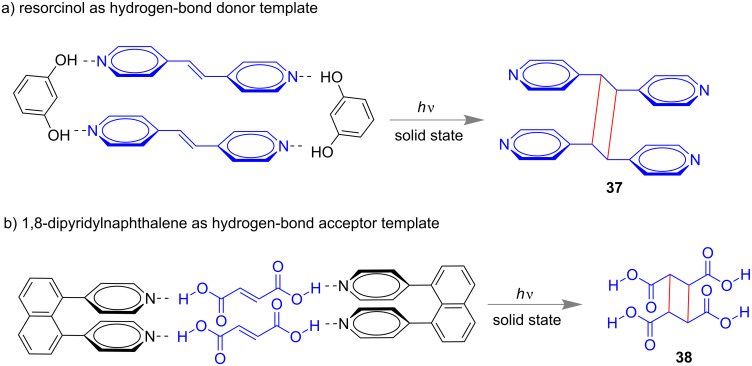
Hydrogen-bond-assisted [2 + 2]-cycloaddition reaction through solid-state grinding. Hydrogen-bond donors are a) resorcinol and b) 1,8-dipyridylnaphthalene, respectively.

It was long believed that covalent-bond formation in supramolecular chemistry which occurs in solution-phase synthesis is almost impossible in solid-state reactions. However, MacGillivray’s group demonstrated several examples of co-crystal formation or supramolecular synthesis in the solid phase through mechano-milling or dry grinding. In 2008, they have established a [2 + 2] photodimerization through solid-state grinding either in neat or liquid-assisted conditions [93]. To achieve 100% stereospecific products they considered resorcinol derivatives as hydrogen-bond donors for the photodimerization of 1,2-di(pyridin-4-yl)ethylene (Figure 21a). However, 1,8-dipyridylnaphthalene was used as hydrogen-bond acceptor for the [2 + 2] cycloaddition of fumaric acid derivatives (Figure 21b).

**Figure 22:**
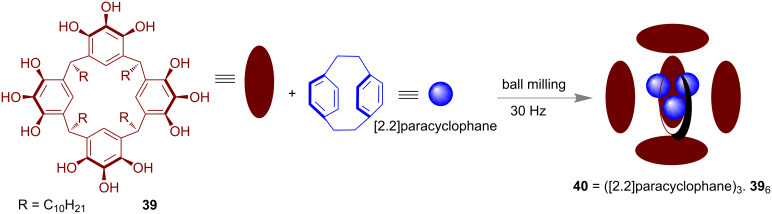
Formation of the cage and encapsulation of [2.2]paracyclophane guest molecule in the cage was done simultaneously under mechanochemical conditions.

In 2017 Purse and co-workers reported the host–guest chemistry of pyrogallo[4]arene (**39**) hexamers under mechano-milling conditions [94]. A hexameric capsule **40** formed through hydrogen-bonding and the cavity was found to be able to encapsulate different organic molecules such as alkanes, acids, amines, etc. The encapsulation of a [2.2]paracyclophane in the cage was achieved by ball milling at 30 Hz (Figure 22) and the host–guest product **40** was verified by NMR as well as other spectroscopic techniques.

**Figure 23:**
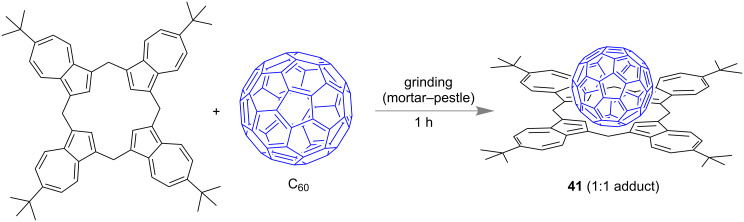
Formation of the 1:1 complex C_60_–*tert*-butylcalix[4]azulene through mortar and pestle grinding of the host and the guest. The structure of the complex was obtained from DFT study.

Georghiou et al. demonstrated the mechanochemical formation of a 1:1 supramolecular complex C_60_–*tert*-butylcalix[4]azulene **41** (Figure 23). The host–guest complexation was achieved by simple grinding the individual compounds in a mortar and pestle [95].

### Supramolecular catalysis

The concept of supramolecular catalysis mainly is based on the use of supramolecular chemistry, molecular recognition, host–guest chemistry, etc. for catalysis [96]. The field originated with the understanding of enzymatic system which is conceptually different from traditional organic chemistry reactions, as it relies on soft force [97–98] or non-covalent interactions [2] such as hydrogen bonding [99], cation–π [100–102], anion–π [103], hydrophobic effect [104–105], halogen bonding [106–109], etc. As enzymes are structurally complex entities and are difficult to modify, supramolecular catalysis proposes a much simpler model to understand the catalytic activity of enzymes.

**Figure 24:**
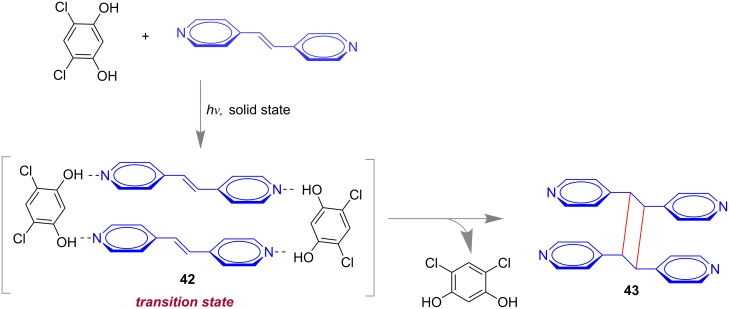
Formation of a 2:2 complex between the supramolecular catalyst and the reagent in the transition state of the [2 + 2]-cycloaddition reaction of 1,2-di(pyridin-4-yl)ethylene and 4,6-dichlororesorcinol.

In 2010, MacGillivray and co-workers have demonstrated the concept of “supramolecular catalysis” in a hydrogen-bond-assisted self-assembled formation of a [2 + 2]-cycloaddition product. The reaction was found to be 100% stereospecific under dry mortar and pestle grinding [110]. The hydrogen-bond donor 4,6-dichlororesorcinol was used as the supramolecular catalyst for the transformation in the solid-state. From single crystal X-ray analysis the authors have proved the formation of the 2:2 complex **42** from 1,2-di(pyridin-4-yl)ethylene and 4,6-dichlororesorcinol in the transition state (Figure 24). Finally the cyclobutane derivative **43** was observed after the release of catalyst for the next cycle.

In 2012, again the MacGillivray group reported an improved version of the above mentioned [2 + 2]-cycloaddition methodology. They used the vertex grinding technique where solid-state grinding and UV irradiation was done simultaneously [111] and verified co-crystal formation of a resorcinol derivative with dipyridylethylene in the solid state. Also, the supramolecular catalysis of [2 + 2] photodimerization has been shown to proceed with excellent turnover numbers.

**Figure 25:**
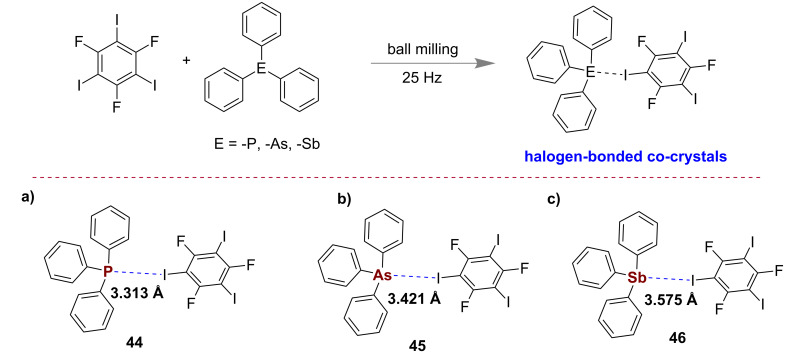
Halogen-bonded co-crystals via a) I···P, b) I···As, and c) I···Sb bonds [112].

Recently, Friščić and Cinčić with co-workers reported an elaborative study on the halogen bonding between 1,3,5-trifluoro-2,4,6-triiodobenzene and triphenylphosphine, -arsine, and -stibine under neat mechanochemical conditions or through solvent-assisted grinding using ethanol (Figure 25). The single crystal X-ray structures of the obtained co-crystals **44**–**46** were reported to match with the solution-phase co-crystals. They have also studied energy levels, thermal properties and the stability of these structures through DFT calculations [112]. In this work they have also demonstrated that metallic pnictogens do form sufficiently strong halogen bonds to enable co-crystal formation.

**Figure 26:**
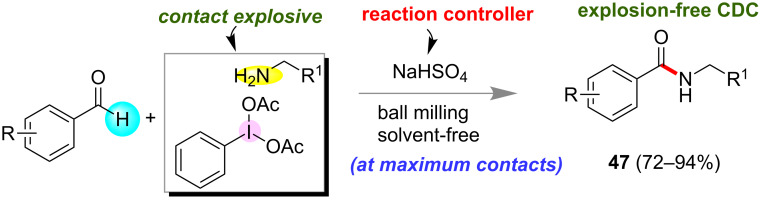
Transformation of contact-explosive primary amines and iodine(III) into a successful chemical reaction for amide synthesis.

Mal and co-workers have shown that a contact explosive, i.e., the mixture of primary amines and phenyliodine diacetate led to a high-yielding reaction at maximum contact (solvent-free ball milling) of the reactants [113]. An acid salt, (sodium bisulfate) was used to control the reactivity of the highly basic primary amines to transform the exceedingly exothermic reactive substrates in a high-yielding cross-dehydrogenative coupling (CDC) reaction to obtain the amides **47** (Figure 26).

**Figure 27:**
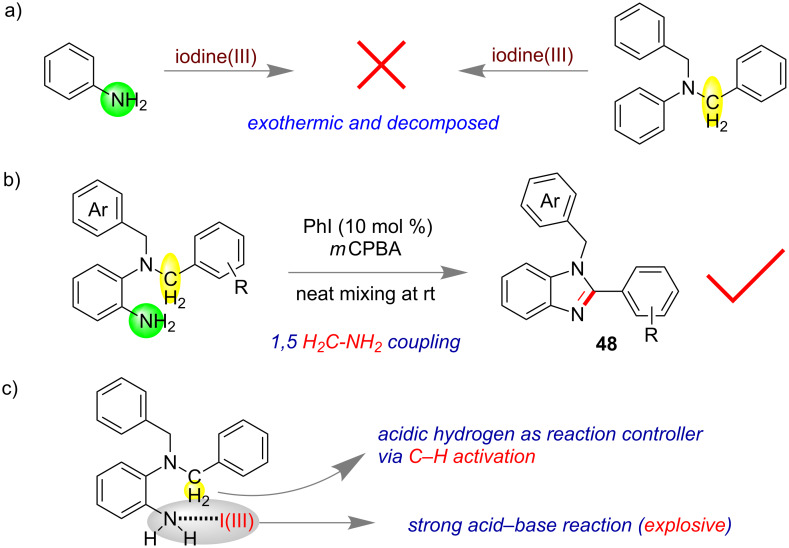
Undirected C–H functionalization by using the acidic hydrogen to control basicity of the amines [114]. a) Identified exothermic reactions. b) Successful reaction by quenching the heat intramolecularly. c) The plausible mechanism of acidic C–H functionalization intramolecularly.

The development of sustainable methods for the activation of less-reactive undirected C(sp^3^)–H bonds is challenging however, highly desired in organic synthesis. Mal and co-workers also demonstrated that acidic C(sp^3^)–hydrogen bonds within a molecule could be used to control exothermic reactions between amines and iodine(III) [114]. By this process undirected C(sp^3^)–H bonds were shown to be functionalized for dehydrogenative imination reactions. Overall, at 1,5-distances (remote) a dehydrogenative and intramolecular C(sp^3^)–H imination by 4H elimination was readily done via organocatalysis using PhI (10 mol %)–*m*CPBA at ambient conditions as well as under neat mixing [115]. The *N*^1^*,N*^1^-dibenzylbenzene-1,2-diamine (Figure 27) which is an integrated system by the combination of aniline and *N,N*-dibenzylaniline led to the successful formation of 1-benzyl-2-phenyl-benzo[*d*]imidazole **48** under the iodine(III) environment.

### Conclusion

Over the last years, substantial progress has been made in the area of mechanochemistry as environmentally benign method in organic synthesis, materials science and supramolecular chemistry. In this review the major focus has been to cover the concept and application of mechanochemistry in the formation of self-assembled supramolecules. In addition, we have included mechanochemical approaches to areas such as subcomponent self-assembly, dynamic combinatorial chemistry, systems chemistry, and supramolecular catalysis. We anticipate that the research area of supramolecular mechanochemistry is still in its infancy and needs significant improvement towards understanding and development of suitable methods [116–118].
